# Supplementary material for: Conserved enhancers control notochord expression of vertebrate Brachyury
Source: Nat Commun. 2023 Oct 18;14:6594. doi: 10.1038/s41467-023-42151-3 (PMC10584899; doi:10.1038/s41467-023-42151-3)
Supplement: Supplementary file 1 — Reporting Summary [file 41467_2023_42151_MOESM1_ESM.pdf]

nature portfolio

Corresponding author(s): Alexa Burger, PhD
Zbynek Kozmik, PhD

Last updated by author(s): 09/25/2023

Reporting Summary

Nature Portfolio wishes to improve the reproducibility of the work that we publish. This form provides structure for consistency and transparency in reporting. For further information on Nature Portfolio policies, see our Editorial Policies and the Editorial Policy Checklist.

Please do not complete any field with "not applicable" or n/a. Refer to the help text for what text to use if an item is not relevant to your study.
For final submission: please carefully check your responses for accuracy; you will not be able to make changes later.

Statistics

For all statistical analyses, confirm that the following items are present in the figure legend, table legend, main text, or Methods section.

n/a	Confirmed	
[ ]	[X]	The exact sample size (n) for each experimental group/condition, given as a discrete number and unit of measurement
[ ]	[X]	A statement on whether measurements were taken from distinct samples or whether the same sample was measured repeatedly
[ ]	[X]	The statistical test(s) used AND whether they are one- or two-sided<br>*Only common tests should be described solely by name; describe more complex techniques in the Methods section.*
[X]	[ ]	A description of all covariates tested
[X]	[ ]	A description of any assumptions or corrections, such as tests of normality and adjustment for multiple comparisons
[X]	[ ]	A full description of the statistical parameters including central tendency (e.g. means) or other basic estimates (e.g. regression coefficient) AND variation (e.g. standard deviation) or associated estimates of uncertainty (e.g. confidence intervals)
[ ]	[X]	For null hypothesis testing, the test statistic (e.g. F, t, r) with confidence intervals, effect sizes, degrees of freedom and P value noted<br>*Give P values as exact values whenever suitable.*
[X]	[ ]	For Bayesian analysis, information on the choice of priors and Markov chain Monte Carlo settings
[X]	[ ]	For hierarchical and complex designs, identification of the appropriate level for tests and full reporting of outcomes
[X]	[ ]	Estimates of effect sizes (e.g. Cohen's d, Pearson's r), indicating how they were calculated

Our web collection on statistics for biologists contains articles on many of the points above.

Software and code

Policy information about availability of computer code

Data collection	BLASTN searches (search sensitivity: distant homologies) were performed at Ensembl.org
Data analysis	JASPAR (https://jaspar.genereg.net/) and FIMO (https://meme-suite.org/meme/tools/fimo) software was used for data analysis.

For manuscripts utilizing custom algorithms or software that are central to the research but not yet described in published literature, software must be made available to editors and reviewers. We strongly encourage code deposition in a community repository (e.g. GitHub). See the Nature Portfolio guidelines for submitting code & software for further information.

Data

Policy information about availability of data

All manuscripts must include a data availability statement. This statement should provide the following information, where applicable:
- Accession codes, unique identifiers, or web links for publicly available datasets
- A description of any restrictions on data availability
- For clinical datasets or third party data, please ensure that the statement adheres to our policy

The authors declare that all the data/databases/datasets supporting the findings of this study are available
- within the paper in the Data availability section and Supplementary information files;
- in a publicly accessible repository using UCSC browser (https://genome.ucsc.edu/);
- from the corresponding authors upon reasonable request (plasmids, stable transgenic zebrafish lines, mouse knockout lines).

Research involving human participants, their data, or biological material

Policy information about studies with human participants or human data. See also policy information about sex, gender (identity/presentation), and sexual orientation and race, ethnicity and racism.

Reporting on sex and gender	N/A
Reporting on race, ethnicity, or other socially relevant groupings	N/A
Population characteristics	N/A
Recruitment	N/A
Ethics oversight	N/A

Note that full information on the approval of the study protocol must also be provided in the manuscript.

Field-specific reporting

Please select the one below that is the best fit for your research. If you are not sure, read the appropriate sections before making your selection.

[X] Life sciences [ ] Behavioural & social sciences [ ] Ecological, evolutionary & environmental sciences

For a reference copy of the document with all sections, see nature.com/documents/nr-reporting-summary-flat.pdf

Life sciences study design

All studies must disclose on these points even when the disclosure is negative.

Sample size	All experimental sample sizes were chosen by common standards in the field and in accordance with solid phenotype designation.
Data exclusions	None.
Replication	Imaging, transgenic, and mutant analysis experiments were treated with identical experimental conditions and performed at least twice and three or more times in the majority of instances and all attempts at replication were successful.
Randomization	Data analyses for transgenic and mutant quantification was based on injections and defined genotypes of zebrafish, axolotl, Ciona and mouse crosses. No other randomizations are applicable.
Blinding	Data collection for transgenic and mutant analyses was unblinded as it required reporter activity an phenotype assessment as well as genotyping analysis to confirm transgenic or mutant versus wildtype.

Behavioural & social sciences study design

All studies must disclose on these points even when the disclosure is negative.

Study description	
Research sample	
Sampling strategy	
Data collection	
Timing	
Data exclusions	
Non-participation	
Randomization	

Ecological, evolutionary & environmental sciences study design

All studies must disclose on these points even when the disclosure is negative.

Study description	
Research sample	
Sampling strategy	
Data collection	
Timing and spatial scale	
Data exclusions	
Reproducibility	
Randomization	
Blinding	

Did the study involve field work? ☐ Yes ☐ No

Field work, collection and transport

Field conditions	
Location	
Access & import/export	
Disturbance	

Reporting for specific materials, systems and methods

We require information from authors about some types of materials, experimental systems and methods used in many studies. Here, indicate whether each material, system or method listed is relevant to your study. If you are not sure if a list item applies to your research, read the appropriate section before selecting a response.

Materials & experimental systems

n/a	Involved in the study
☐	☒ Antibodies
☒	☐ Eukaryotic cell lines
☒	☐ Palaeontology and archaeology
☐	☒ Animals and other organisms
☒	☐ Clinical data
☒	☐ Dual use research of concern
☒	☐ Plants

Methods

n/a	Involved in the study
☒	☐ ChIP-seq
☒	☐ Flow cytometry
☒	☐ MRI-based neuroimaging

Antibodies

Antibodies used

mouse anti-Fibronectin (ab6328, Abcam; 1:400) and donkey anti-mouse-Alexa 568 (A10037, Invitrogen; 1:600)

rabbit anti-Brachyury (ab209665, Abcam, 1:2000) and donkey anti-rabbit Alexa Fluor™ 594 (A-11011, Invitrogen, 1:500).

goat anti-Sox2 Y-17 (sc-17320, Santa Cruz), dilution 1:400, and donkey anti-goat Alexa Fluor™ 488 (A-11055, Invitrogen, 1:500)

Validation

These antibodies are standards in the field and were validated according to the manufacturer's instructions.

Eukaryotic cell lines N/A

Policy information about cell lines and Sex and Gender in Research

Cell line source(s)

Authentication

Mycoplasma contamination

Commonly misidentified lines
(See ICLAC register)

Palaeontology and Archaeology N/A

Specimen provenance

Specimen deposition

Dating methods

☐ Tick this box to confirm that the raw and calibrated dates are available in the paper or in Supplementary Information.

Ethics oversight

Note that full information on the approval of the study protocol must also be provided in the manuscript.

Animals and other research organisms

Policy information about studies involving animals; ARRIVE guidelines recommended for reporting animal research, and Sex and Gender in Research

Laboratory animals	Age of adult animals was according to each species minimal breeding age. Further used were<br>* Zebrafish embryos (50-80% epiboly, 24 hpf, 48 hpf) from adult (3 months old) AB and TU wildtype strains, as well as adult (3 months old) transgenics of mixed background<br>* Stage 21 Ciona robusta embryos, derived from commercially obtained adults (2-3 months old)<br>* Stage 14-43 Axolotl embryos, from White mutant (d/d) adults (1 year old)<br>* E9.5 and 12.5 mouse embryos from adult FVB, CD-1. C57BL/6N mice (6-8 weeks old)
Wild animals	No wild animals were used in the study.
Reporting on sex	Zebrafish and axolotl embryos were not selected by gender as sex determination happens later in development. Ciona are hermaphroditic, therefore there is only one possible sex for individuals. No differences in gender were observed in the transgenic or knockout studies in mouse embryos.
Field-collected samples	No field collected samples were used in the study.
Ethics oversight	* Zebrafish animal care and procedures were carried out in accordance with the IACUC of the University of Colorado Anschutz Medical Campus (protocol # 00979), Aurora, Colorado.<br>* Axolotl husbandry and experiments (non-free feeding stages) were performed in compliance with the laws and regulations of the State of Saxony, Germany, at the CRTD/Center for Regenerative Therapies Dresden, Dresden, Germany and do not need ethics approval at the stages used here.<br>* Ciona experiments were performed at UCSD and do not need ethics approval due to the non-vertebrate character of the species.<br>* Transgenic mouse assays were conducted at the E.O. Lawrence Berkeley National Laboratory (LBNL) and performed under U.S. Department of Energy Contract DE-AC02-05CH11231, University of California (UC), and animal protocol number 290003; reviewed and approved by the Animal Welfare and Research Committee at Lawrence Berkeley National Laboratory.<br>* Mouse knockout procedures and animal care were approved by the Animal Care Committee of the Institute of Molecular Genetics (IMG), Czech Academy of Sciences, Prague, Czech Republic, and covered under protocol permission number 357/2021. Experiments were performed in compliance with the European Communities Council Directive of November 24, 1986 (86/609/EEC), as well as national and institutional guidelines.

Note that full information on the approval of the study protocol must also be provided in the manuscript.

Clinical data N/A

Policy information about clinical studies

All manuscripts should comply with the ICMJE guidelines for publication of clinical research and a completed CONSORT checklist must be included with all submissions.

Clinical trial registration

Study protocol

Data collection

Outcomes

Dual use research of concern

Policy information about dual use research of concern

Hazards

Could the accidental, deliberate or reckless misuse of agents or technologies generated in the work, or the application of information presented in the manuscript, pose a threat to:

No	Yes	
X		Public health
X		National security
X		Crops and/or livestock
X		Ecosystems
X		Any other significant area

Experiments of concern

Does the work involve any of these experiments of concern:

No	Yes	
X		Demonstrate how to render a vaccine ineffective
X		Confer resistance to therapeutically useful antibiotics or antiviral agents
X		Enhance the virulence of a pathogen or render a nonpathogen virulent
X		Increase transmissibility of a pathogen
X		Alter the host range of a pathogen
X		Enable evasion of diagnostic/detection modalities
X		Enable the weaponization of a biological agent or toxin
X		Any other potentially harmful combination of experiments and agents

Plants N/A

Seed stocks	
Novel plant genotypes	
Authentication	

ChIP-seq N/A

Data deposition

- [ ] Confirm that both raw and final processed data have been deposited in a public database such as GEO.
- [ ] Confirm that you have deposited or provided access to graph files (e.g. BED files) for the called peaks.

Data access links<br>*May remain private before publication.*	
Files in database submission	
Genome browser session<br>(e.g. UCSC)	

Methodology

Replicates	
Sequencing depth	
Antibodies	
Peak calling parameters	
Data quality	
Software	

Flow Cytometry N/A

Plots

Confirm that:

☐ The axis labels state the marker and fluorochrome used (e.g. CD4-FITC).

☐ The axis scales are clearly visible. Include numbers along axes only for bottom left plot of group (a 'group' is an analysis of identical markers).

☐ All plots are contour plots with outliers or pseudocolor plots.

☐ A numerical value for number of cells or percentage (with statistics) is provided.

Methodology

Sample preparation

Instrument

Software

Cell population abundance

Gating strategy

☐ Tick this box to confirm that a figure exemplifying the gating strategy is provided in the Supplementary Information.

Magnetic resonance imaging N/A

Experimental design

Design type

Design specifications

Behavioral performance measures

Imaging type(s)

Field strength

Sequence & imaging parameters

Area of acquisition

Diffusion MRI ☐ Used ☐ Not used

Preprocessing

Preprocessing software

Normalization

Normalization template

Noise and artifact removal

Volume censoring

Statistical modeling & inference

Model type and settings

Effect(s) tested

Specify type of analysis: ☐ Whole brain ☐ ROI-based ☐ Both

Statistic type for inference

(See Eklund et al. 2016)

Correction

Models & analysis

n/a	Involved in the study
☐	☐ Functional and/or effective connectivity
☐	☐ Graph analysis
☐	☐ Multivariate modeling or predictive analysis

Functional and/or effective connectivity

Graph analysis

Multivariate modeling and predictive analysis

This checklist template is licensed under a Creative Commons Attribution 4.0 International License, which permits use, sharing, adaptation, distribution and reproduction in any medium or format, as long as you give appropriate credit to the original author(s) and the source, provide a link to the Creative Commons license, and indicate if changes were made. The images or other third party material in this article are included in the article's Creative Commons license, unless indicated otherwise in a credit line to the material. If material is not included in the article's Creative Commons license and your intended use is not permitted by statutory regulation or exceeds the permitted use, you will need to obtain permission directly from the copyright holder. To view a copy of this license, visit http://creativecommons.org/licenses/by/4.0/
