# Supplementary material for: Conserved enhancers control notochord expression of vertebrate Brachyury
Source: Nat Commun. 2023 Oct 18;14:6594. doi: 10.1038/s41467-023-42151-3 (PMC10584899; doi:10.1038/s41467-023-42151-3)
Supplement: Supplementary file 2 — Supplementary Information [file 41467_2023_42151_MOESM2_ESM.pdf]

Supplementary Information to the manuscript: Conserved enhancers control notochord expression of vertebrate Brachyury by Kemmler et al.

This Supplementary Information contains information about the Supplementary Data 1-7, as well as Supplementary Figures 1-6. Supplementary Data 1-6 are excel tables, Supplementary Data 7 are MAFFT alignments and sequence files compiled as a .zip file.

Supplementary Data 1: Genomic features of the human enhancer elements.
Summary table listing the genomic features of the human enhancer elements, including length, location relative to transcription start (TS) site, ATAC- or T ChIP-seq peaks, conservation in mouse and *Monodelphis*, H3K27ac, and ENCODE cCREs.

Supplementary Data 2: Reporter activity across animal models.
All numbers from the enhancer reporter experiments in zebrafish, axolotl, mouse, and Ciona.

Supplementary Data 3: Coordinates of all cloned enhancer elements.
Summary table displaying the genomic coordinates of all enhancer elements from different species, as well as primer sequences used to amplify them, length, and reporter activity of the enhancers in the different species.

Supplementary Data 4: *Tbxtb* enhancer element conservation across vertebrates.
Genomic location and genome versions are provided for each species. BLAST bridging chain is indicated with -> showing BLAST hits from *Tbxtb* loci of one species to another and -x indicating lack chaining. (2x) indicate tetraploid species with up to two *tbxtb* loci.

Supplementary Data 5: Enhancer element deletions and primer sequences for genotyping.
Summary table with genomic coordinates and sequences of the used target sites, primer, and sequences of the three enhancer deletions.

Supplementary Data 6: Qualitative evaluation of Brachyury antibody staining in E9.5 embryos.
Summary table of qualitative evaluation of anti-Brachyury/T staining in E9.5 embryos.

Supplementary Data 7: Sequence and alignment files of *T3*, *C*, and *I* for Fig. 6 and Supplementary Fig. 6

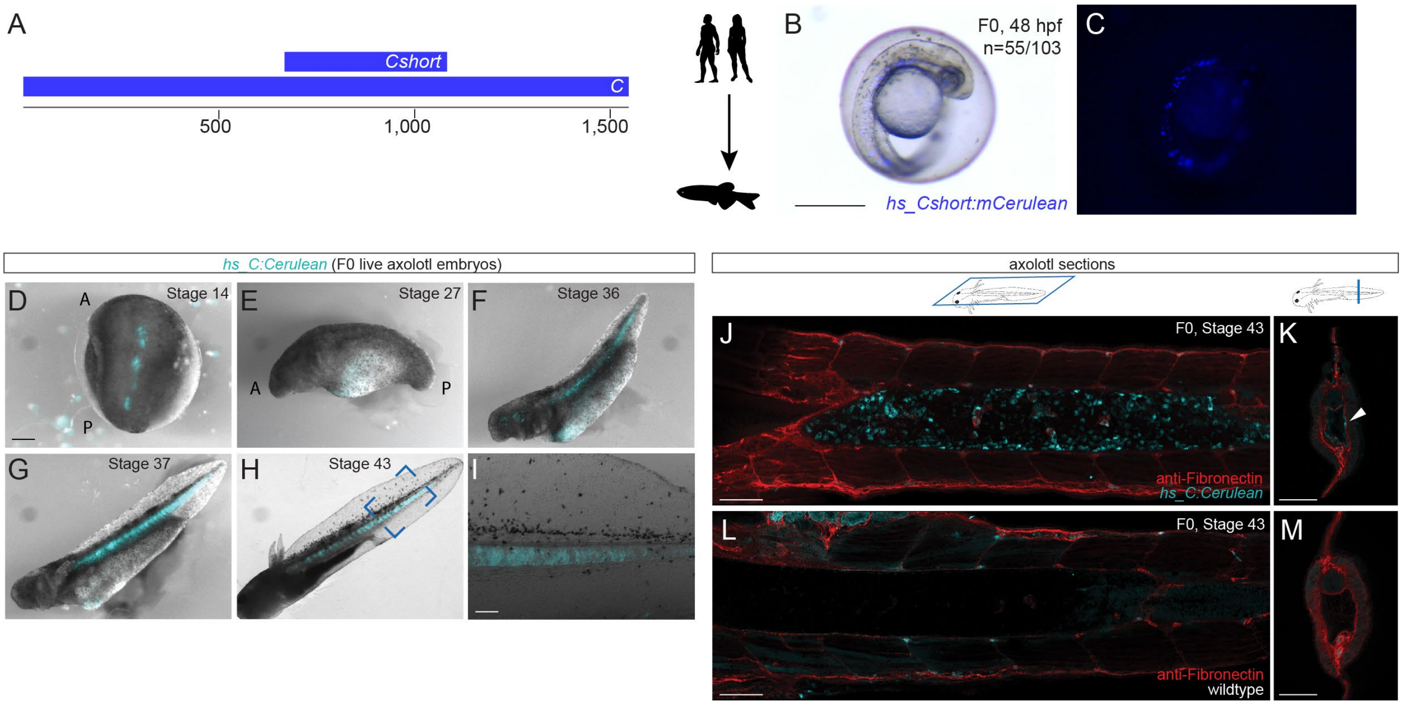

Supplementary Figure 1: Human enhancer element *Cshort* in zebrafish and time course of human enhancer element *C* in axolotl.

(**A**) Schematic representation of human enhancer element *C* and *Cshort*.

(**B,C**) Representative F0 transgenic zebrafish embryo expressing *hs_Cshort:mCerulean* in the notochord at 2 dpf. Images shown are a merge of bright field and fluorescence (**B**) and fluorescence only (**C**). Scale bar in **B**: 0.5 mm, applies to **B**,**C**.

(**D-I**) Live images of representative F0 transgenic axolotl embryos expressing *hs_C:mCerulean* at stages 14 (**D**), 27 (**E**), 36 (**F**), 37 (**G**), 43 (**H**) and close up of **H** from the blue outline (**I**). (**D**) dorsal view. (**D,E**) A, anterior, P, posterior. Images shown are a merge of bright field and fluorescence. Scale bar in **D**: 1 mm; applies to panels **D-H**. Scale bar in **I**: 0.5 mm.

(**J-M**) Confocal images of horizontal (**J,L**) and cross (**K,M**) sections through the axolotl embryo (stage 43) show mCerulean fluorescence in the notochord in transgenic *hs_C:mCerulean* embryos (**J,K**) compared to wildtype embryos (**L,M**), but not in the surrounding muscle which is highlighted by immunostaining of fibronectin in red. Scale bars in **J-M**: 0.5 mm. The species silhouettes were adapted from the PhyloPic database (www.phylopic.org).

A

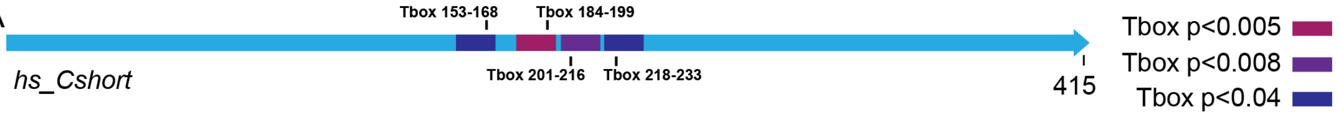

B

Motif ID	Name	Input	Length	Start	Stop	Strand	Score	P value	Q value	Matched sequence
MA0009.2	TBXT	*hs_C*	415 bp	153	168	+	-20.2115	0.0341	1	TCATCCACATCTGCAA
MA0009.2	TBXT	*hs_C*	415 bp	153	168	-	-8.5	0.00532	0.995	TTGCAGATGTGGATGA
MA0009.2	TBXT	*hs_C*	415 bp	218	233	+	-10.0577	0.00709	0.995	AAGCAAACACAGGAGA
MA0009.2	TBXT	*hs_C*	415 bp	218	233	-	-21.1154	0.0385	1	TCTCCTGTGTTTGCTT

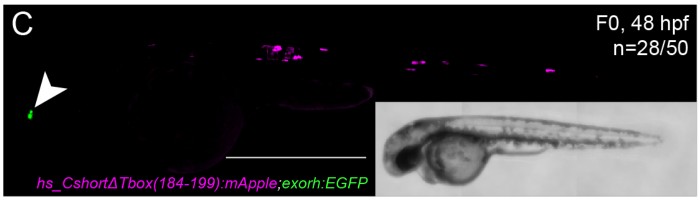

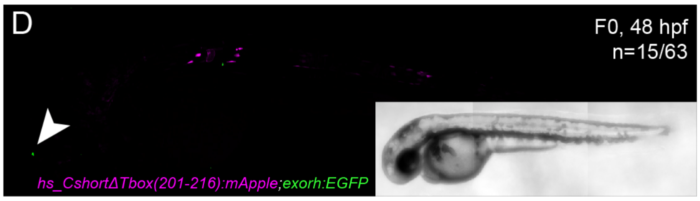

Supplementary Figure 2: Additional identified TBXT binding sites in enhancer *C*.
(**A**) Schematic depiction of the human enhancer element *hs_Cshort* including the four TBXT binding sites/T-box motifs with different p-values; reverse complement direction. P values were calculated by FIMO.
(**B**) FIMO output with location of the T-box motifs, statistical significance, and matched sequence within the enhancer elements.
(**C,D**) Injection of the enhancer element *hs_Cshort* with individual deleted T-box motifs *hs_CshortΔTbox(184-199)* (**C**) and *hs_CshortΔTbox(201-216)* (**D**) as reporter constructs results in maintained reporter activity. Arrowheads (**C,D**) mark EGFP expression in the pineal gland from the transgenesis marker *exorh:EGFP*. Scale bar in **C**: 0.5 mm, applies to **C,D**.

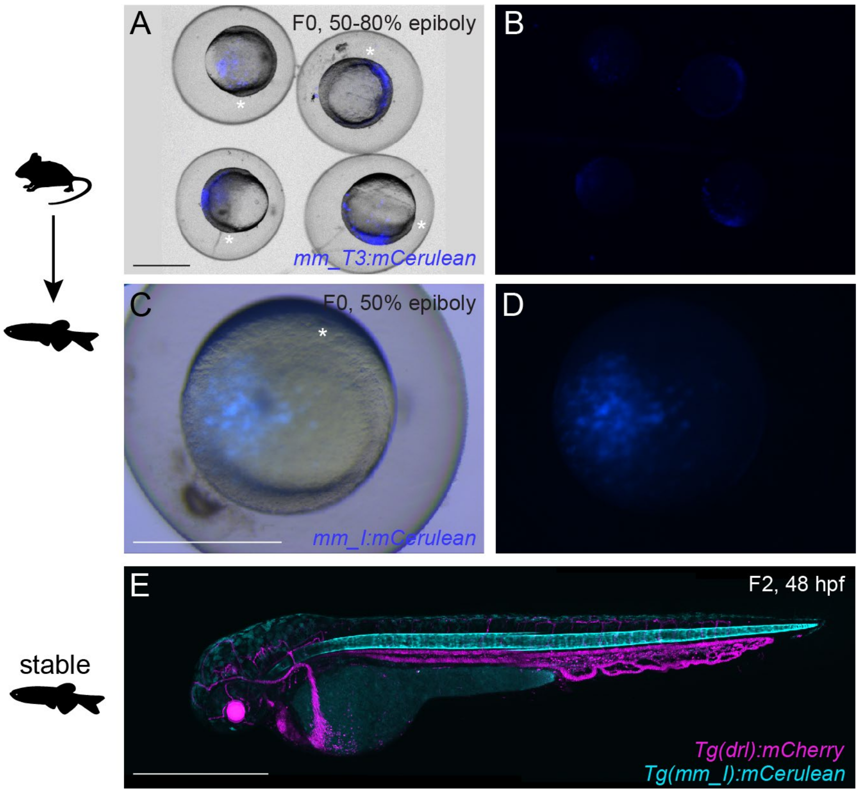

Supplementary Figure 3: Mouse enhancer elements *T3* and *I* at 80% epiboly in zebrafish and mouse enhancer element *I* stable zebrafish line.

(**A-D**) Mouse enhancer element *mm_T3* and *mm_I* in zebrafish at 80% epiboly. Live images of representative F0 transgenic zebrafish embryos expressing *mm_T3:mCerulean* in the zebrafish embryo at 80% epiboly. Images shown are a merge of bright field and fluorescence (**A**) and fluorescence only (**B**). Further, live images of a representative F0 transgenic zebrafish embryo expressing *mm_I:mCerulean* in the zebrafish embryo at 80% epiboly. Images shown are a merge of bright field and fluorescence (**C**) and fluorescence only (**D**). Scale bars in **A**,**C**: 0.5 mm, applies to **A**,**B** and **C**,**D**. Asterisks in **A, C** mark the shield.

(**E**) Representative image of a F2 embryo at 2 dpf from F1 stable line for mouse enhancer element *I* crossed to *Tg(drl:mCherry)* stable line labelling lateral plate mesoderm lineages. Transgenic F2 embryo recapitulates the F0 expression pattern in the notochord. Scale bar in **E**: 0.5 mm. The species silhouettes were adapted from the PhyloPic database (www.phylopic.org).

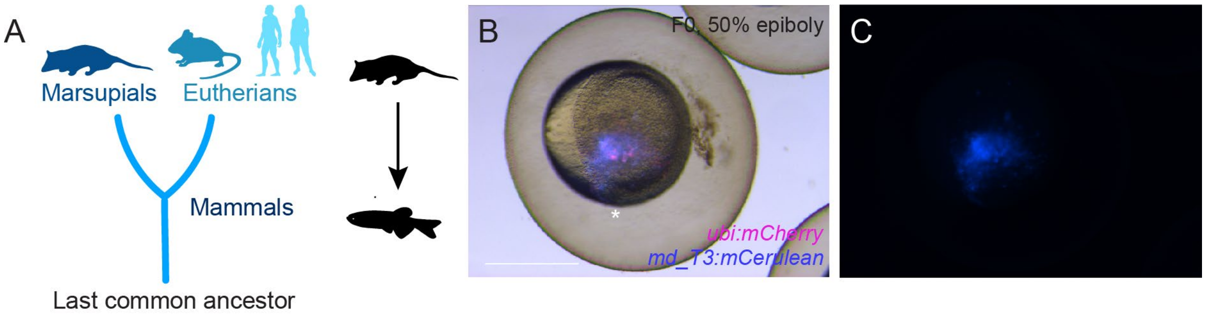

Supplementary Figure 4: Additional data to *Monodelphis domestica* enhancer elements.
(**A**) Mammalian phylogeny outlining the split into Marsupials and Eutherians.
(**B,C**) Monodelphis enhancer element *md_T3* in zebrafish at 80% epiboly. Live images of representative F0 transgenic zebrafish embryos expressing *md_T3:mCerulean* and *ubi:mCherry* in the zebrafish embryo at 80% epiboly. Images shown are a merge of bright field and fluorescence (**B**) and fluorescence only (**C**). Scale bar in **B**: 0.5 mm, applies to **C**. Asterisk in **B** marks the shield. The species silhouettes were adapted from the PhyloPic database (www.phylopic.org).

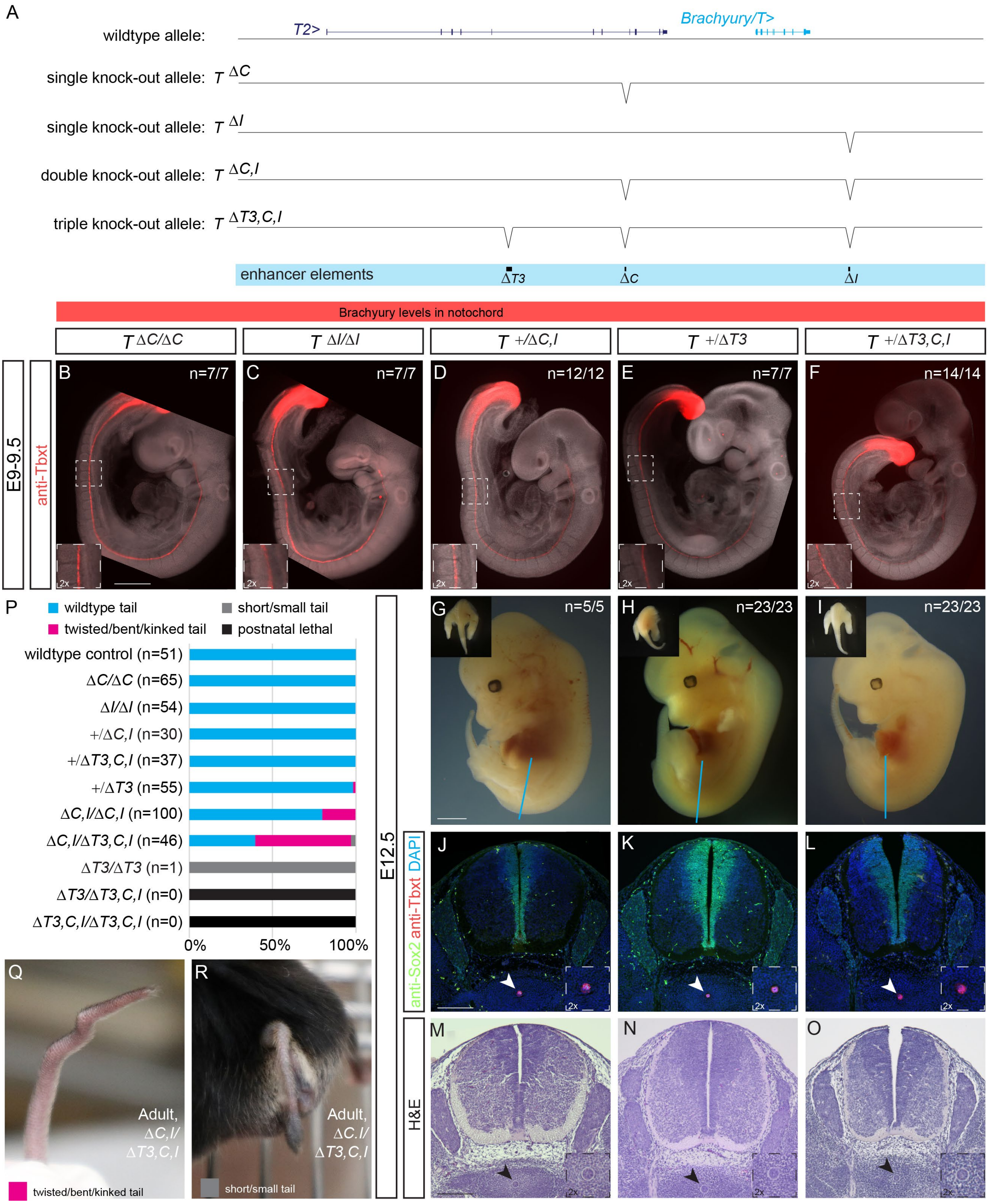

Supplementary Figure 5: Additional data to deletion of the three enhancer elements.
(**A**) Mouse *Brachyury/T/TBXTB* locus adapted from UCSC browser and annotation of single knockout alleles *ΔC* and *ΔI*.
(**B-F**) E9.5 homozygous *ΔC* (**B**), homozygous *ΔI* embryos (**C**), heterozygous *+/ΔC,I* (**D**), heterozygous *+/ΔT3* (**E**), and heterozygous *+/ΔT3,C,I* embryos (**F**) display normal Brachyury/T protein expression (red) in the notochord as depicted by anti-T immunofluorescence. White dashed square in panels represents location of right bottom inserts with 2x magnification. Scale bar in **B**: 1 mm, applies to panels **B-F**.
(**G-I**) Overall morphology of E12.5 embryos with different genotypes. Inserts in the left upper corner represent

anterior view of the trunk and tails. Blue lines indicate the location of immunofluorescence and H&E sections. Inserts in the top left indicate wildtype looking tails. Scale bar in **G**: 1 mm, applies to panels **G-I**.
(**J-L**) Immunofluorescence of mouse transverse sections. Anti-Sox2 labels the neural plate, anti-Tbxt the notochord, and DAPI marks nuclei. Sox2 and Brachyury/T expression is comparable amongst the shown genotypes. Scale bar in **J**: 0.2 mm, applies to **J-L**.
(**M-O**) H&E staining of transverse sections confirm normal notochords. Arrowheads point to notochord. Scale bar in **M**: 0.2 mm, applies to **M-O**.
(**P**) Percentage of adult animals with tail phenotypes.
(**Q,R**) Representative images of the kinked and small tail phenotype *in* $T^{\Delta C,I/\Delta T3,C,I}$ *trans*-heterozygous adult animals.

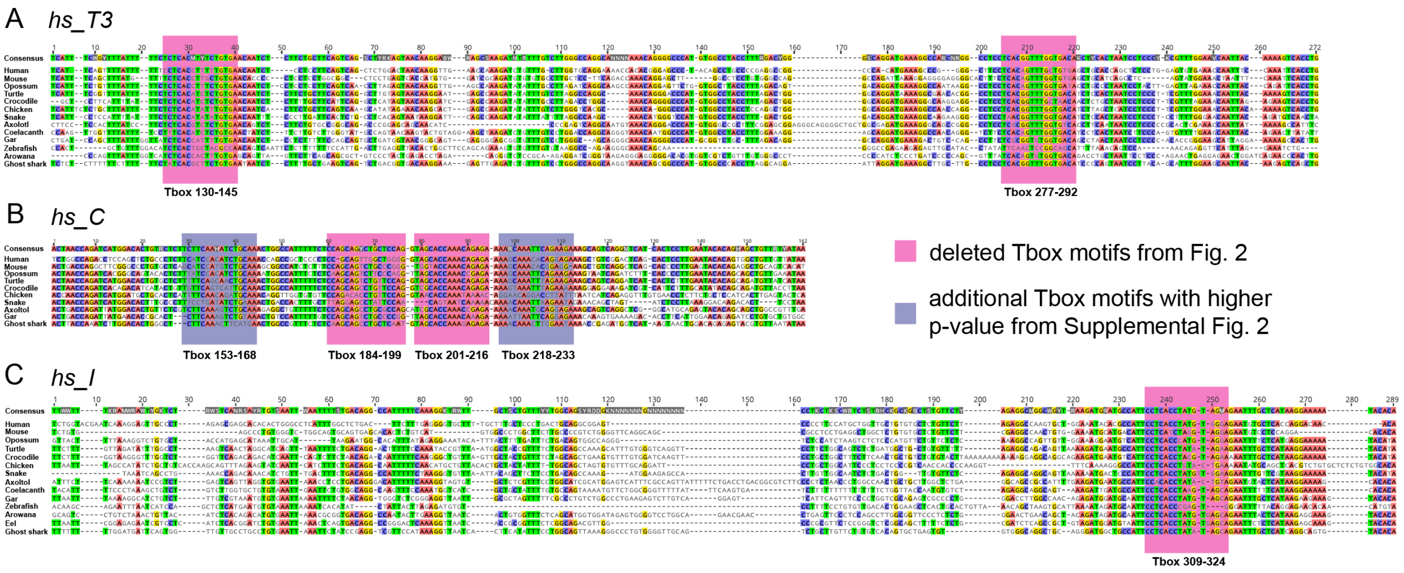

Supplementary Figure 6: Conservation of the enhancers *T3*, *C*, and *I* with their respective T-boxes. (**A-C**) Conservation of the enhancers *T3* (**A**), *C* (**B**), and *I* (**C**) and respective T-box motifs in different species. T-box motifs with low p-value are marked in pink (see **Fig. 2**) and T-box motifs with higher p-value are marked in violet (see **Supplementary Fig. 2**).
