# Supplementary material for: Conserved enhancers control notochord expression of vertebrate Brachyury
Source: Nat Commun. 2023 Oct 18;14:6594. doi: 10.1038/s41467-023-42151-3 (PMC10584899; doi:10.1038/s41467-023-42151-3)
Supplement: Supplementary file 4 — Description of Additional Supplementary Files [file 41467_2023_42151_MOESM4_ESM.pdf]

Supplementary Data 1: Genomic features of the human enhancer elements.
Summary table listing the genomic features of the human enhancer elements, including length, location relative to transcription start (TS) site, ATAC- or T ChIP-seq peaks, conservation in mouse and *Monodelphis*, H3K27ac, and ENCODE cCREs.

Supplementary Data 2: Reporter activity across animal models.
All numbers from the enhancer reporter experiments in zebrafish, axolotl, mouse, and Ciona.

Supplementary Data 3: Coordinates of all cloned enhancer elements.
Summary table displaying the genomic coordinates of all enhancer elements from different species, as well as primer sequences used to amplify them, length, and reporter activity of the enhancers in the different species.

Supplementary Data 4: *Tbxtb* enhancer element conservation across vertebrates.
Genomic location and genome versions are provided for each species. BLAST bridging chain is indicated with -> showing BLAST hits from *Tbxtb* loci of one species to another and -x indicating lack chaining. (2x) indicate tetraploid species with up to two *tbxtb* loci.

Supplementary Data 5: Enhancer element deletions and primer sequences for genotyping.
Summary table with genomic coordinates and sequences of the used target sites, primer, and sequences of the three enhancer deletions.

Supplementary Data 6: Qualitative evaluation of Brachyury antibody staining in E9.5 embryos.
Summary table of qualitative evaluation of anti-Brachyury/T staining in E9.5 embryos.

Supplementary Data 7: Sequence and alignment files of *T3*, *C*, and *I* for Fig. 6 and Supplementary Fig. 6
